# Trimodal therapy for stage III-N2 non-small-cell lung carcinoma: a single center retrospective analysis

**DOI:** 10.1186/1471-2407-14-572

**Published:** 2014-08-07

**Authors:** Vasileios Askoxylakis, Judith Tanner, Jutta Kappes, Hans Hoffmann, Nils H Nicolay, Harald Rief, Juergen Debus, Michael Thomas, Marc Bischof

**Affiliations:** Department of Radiation Oncology, University of Heidelberg, Heidelberg, Germany; Department of Pulmonary and Respiratory Care Medicine, Thoraxklinik Heidelberg, University of Heidelberg, Heidelberg, Germany; Department of Thoracic Surgery, Thoraxklinik Heidelberg, University of Heidelberg, Heidelberg, Germany; Department of Internal Medicine – Thoracic Oncology, Clinic for Thoracic Diseases, University of Heidelberg, Heidelberg, Germany; Translational Lung Research Center Heidelberg (TLRC-H), Member of the German Center for Lung Research (DZL), Heidelberg, Germany

**Keywords:** NSCLC, Stage III-N2, Trimodal treatment, Radiotherapy

## Abstract

**Background:**

Treatment of locally advanced non-small-cell lung cancer is based on a combined approach. To study the impact of trimodal therapy for stage III-N2 NSCLC a single centre retrospective evaluation focusing on survival and therapy-related toxicity was performed.

**Methods:**

71 patients diagnosed between March 2001 and August 2008 with pathologically confirmed stage III-N2 non-small-cell lung cancer at the University Clinic of Heidelberg were retrospectively analyzed. All patients were treated within trimodal therapy strategies including surgery, induction or adjuvant chemotherapy and postoperative radiotherapy. Overall survival (OS) and disease free survival (DFS) rates were calculated using the Kaplan-Meier method. The log-rank test and Fishers Exact test were applied for univariate analysis and Cox proportional regression model for multivariate analysis.

**Results:**

Median survival was 32 months. 1-, 3- and 5-year overall survival (OS) rates were 84.5%, 49.6% and 35.5% respectively. Disease free survival rates at 1, 3 and 5 years were 70.4%, 41.8% and 27.4% respectively. 9 patients (12.6%) were diagnosed with a local recurrence. Multivariate analysis did not reveal any independent prognostic factors for OS, but indicated a trend for pT stage and type of surgery. In regard to toxicity 8.4% of the patients developed a clinically relevant ≥ grade 2 pneumonitis. Evaluation of the forced expiratory volume in 1 second per unit of vital capacity (FEV1/VC) before and 1-3 years post radiotherapy revealed a median decrease of 2.1%.

**Conclusions:**

Our descriptive data indicate that trimodal therapy represents an effective and safe treatment approach for patients with stage III-N2 non-small-cell lung cancer. Further prospective clinical trials are necessary in order to clearly define the impact of multimodal strategies and optimize NSCLC treatment.

## Background

Lung cancer is the leading cause of cancer mortality in industrialized nations. The disease is diagnosed worldwide in about 1.35 million patients and is responsible for about 1.18 million deaths yearly [1]. Non-small-cell lung cancer (NSCLC) accounts for about 80% of all cases, whereas one-third of the patients are diagnosed with stage III disease, characterized in most cases by involvement of mediastinal lymph nodes (N2). Clinical trials have investigated various therapeutic methods, based on both mono- and multimodal approaches. Surgical resection has a key role for resectable cases, however the prognosis is poor when it is not combined with further modalities. This is mainly attributed to the fact that stage III non-small-cell lung cancer is associated with a high probability for local and distant failure, supporting the thesis that at this stage NSCLC has potentially features of a systemic disease [2]. Furthermore, the complexity of treating stage III NSCLC is strengthened by the high heterogeneity in the patient collective [3].

The poor treatment outcome for stage III NSCLC indicates the necessity for the development of neoadjuvant and/or adjuvant therapeutic strategies and has led in the past decade to numerous clinical trials investigating the efficacy of multimodal approaches. Such approaches focussed mainly on two subsets of patients. The first subset included patients with low tumor burden, considered to be resectable at diagnosis, whereas the second subset consisted of patients with locally advanced tumors that were not considered resectable at diagnosis. In both cases the rationale for the multimodal approaches is based on the optimization of distant disease control through chemotherapy at the possible lowest micrometastatic burden and the optimization of the locoregional control through radiation therapy [4, 5].

The high impact of chemotherapy on treatment outcome has been demonstrated by several trials leading to the establishment of systemic treatment as standard therapy besides surgery. In particular, survival was found to be improved when pre-operative induction chemotherapy was applied to patients with resectable disease compared to surgery alone [6, 7]. Furthermore, a pooled analysis of 5 randomized studies including more than 4,500 patients revealed a 17% reduction in the risk of death for stage III patients receiving cisplatin-based adjuvant chemotherapy [8]. Among prospective phase III trials the Adjuvant Navelbine International Trialist Association (ANITA) study investigated the effects of postoperative chemotherapy in patients with completely resected NSCLC stage IB-IIIA revealing a 5-year overall survival improvement of 8.6%, with a subset analysis demonstrating the highest survival profit for those with stage IIIA disease [9].

However, although the role of chemotherapy has been extensively characterized, there is still an increased need to evaluate the impact of radiotherapy in multimodal treatment settings of stage III NSCLC. A randomized phase III trial investigating concurrent radiotherapy and chemotherapy followed by surgery versus chemotherapy with definitive radiotherapy without surgery, showed a statistically improved progression free survival for the trimodal therapy concept, as well as a trend to improved 5-year overall survival [4]. A further phase III trial investigated the effects of preoperative chemo-radiation in addition to preoperative chemotherapy for patients with NSCLC stage III, revealing an improvement in pathological response and mediastinal downstaging but also an increased post-pneumonectomy mortality [10]. In regard to adjuvant radiotherapy a retrospective analysis of data generated by the ANITA trial revealed an improved 5-year survival for patients with pN2 status who received additional postoperative radiotherapy both in the chemotherapy and the observation arm [11]. However, a large meta-analysis in the past did not reveal the same benefit. The PORT meta-analysis did not show a significant survival benefit for stage III/N2 patients, allowing the conclusion that the role of postoperative radiotherapy in the treatment of N2 tumors is not clear and needs further research [12]. More recent meta-analysis support the hypothesis that modern postoperative radiotherapy may improve local control and survival [13], still further evidence is necessary.

Therefore, aim of the current study is to evaluate the results of a retrospective analysis of 71 patients with stage III-N2 NSCLC, who received trimodal treatment in our institution, including postoperative radiation therapy. The main hypothesis is that trimodal treatment is effective, with acceptable toxicity. Beside overall and disease free survival a subset analysis of treatment outcome and toxicity has been performed in order to generate information that will form the basis for further prospective trials focusing on the role of multimodal approaches in the treatment of resectable stage III-N2 non-small-cell lung cancer.

## Methods

### Patients

71 patients diagnosed between March 2001 and August 2008 with pathologically confirmed stage III-N2 non-small-cell lung cancer and treated within a trimodal approach at the University Hospital of Heidelberg were included in our analysis. N2 status was histologically confirmed in all cases (pN2). Preoperative staging included for all patients CT-scans of the thorax, abdomen and brain, as well as a bone scan. Retrospective evaluation of the patients´ medical records and follow-up data was performed. Analysis included gender, age, histology, tumor site, TNM classification, tumor resection, R-status, chemotherapy, radiotherapy, patterns of treatment failure, disease free survival, overall survival and radiation induced toxicity.

### Surgery

All patients underwent lobectomy, bilobectomy or pneumonectomy. Surgical treatment included a systematic multilevel mediastinal lymph node dissection. Among 44 patients who received lobectomy, 4 (9%) received a sleeve resection.

### Chemotherapy

70 patients received platin-based chemotherapy. Among them 43 received cisplatin-based and 27 carboplatin-based chemotherapy. 1 Patient received gemcitabine monotherapy. 23 patients received preoperative induction chemotherapy with a median of three cycles (range 1-3). 48 patients received postoperative chemotherapy with a median of four cycles (2-4). The decision for neoadjuvant versus adjuvant chemotherapy was an individual decision, based on tumor characteristics, such as tumor size or resectability at diagnosis. The chemotherapy choice (cisplatin versus non-cisplatin) was based on the performance status and co-morbidities, i.e. renal function.

### Radiotherapy

All patients received postoperative radiotherapy (PORT). PORT was applied as three-dimensional conformal radiotherapy (3DCRT). The target volume included mediastinal nodes. Patients with R0 resection received a median dose of 50 Gy (range 50-56 Gy), whereas patients with R1 status received a boost of 10 Gy to a total median dose of 60 Gy (range 40-60 Gy). Patients with primary tumor localization in the upper lobe or involvement of upper mediastinal lymph nodes received individually an irradiation of the supraclavicular fossae to a median total dosis of 50 Gy. Radiation therapy was performed with a linear accelerator at 2 Gy per fraction, 5 days per week.

### Follow up

Patient follow up was performed at 6-8 weeks post radiation treatment and then every 3 months for the first 2 years and thereafter every 6 months. Follow-up included a physical examination and thoracic computed tomography scans, as well as further imaging modalities dependent on the patient´s clinical symptoms. All patients received function tests, including forced expiratory volume in 1 s (FEV_1_) and vital capacity (VC) post surgery but pre-radiotherapy. In 31 cases (44%) post treatment pulmonary function tests were performed at 1-3 years after radiation treatment. Data cut-off was defined as the date of the last follow-up visit at the University Hospital of Heidelberg. Thereafter, only data on survival was obtained by the patient’s physician of choice.

### Data analysis

Overall survival (OS) was defined from the day of treatment begin to the time of death from any cause or last follow up. Disease free survival (DFS) was defined from the day of treatment begin to the day of disease local or distant recurrence, diagnosed by imaging examinations according to the RECIST criteria (Response Evaluation Criteria in Solid Tumours) [14], death or last follow up. Pneumonitis was defined by clinical as well as radiographic findings correlating to the irradiated lung volumes. Pneumonitis was considered clinically relevant (≥grade 2 according to RTOG scale) if persistent cough required antitussive agents or administration of steroids and hospitalization. The ratios of FEV_1_ and VC at 1-3 years post radiotherapy to the respective values before radiotherapy but post surgery were calculated for assessment of radiation induced changes in the pulmonary function. The forced expiratory volume in 1 s per unit of vital capacity (FEV1/VC) was assessed and used as a measure for obstruction.

### Statistics

Statistical analysis was performed using the Statistica version 6.1 Software (StaSoft Inc®, Tulsa OK, USA) and the STATA 13 Data Analysis and Statistical Software. Survival rates were calculated using the Kaplan-Meier method. Subgroup analysis was performed using the log-rank test and Fishers Exact test. Multivariate analysis was performed using a Cox proportional hazards regression model. A p value <0.05 was considered statistically significant.

### Ethics

The study was approved by the ethics committee of the University of Heidelberg, Heidelberg, Germany (S-334/2013).

## Results

### Patient related parameters

Median patient age was 59 years (range, 29-75 years). Median follow-up was 30 months (range, 6-93 months). Histology analysis revealed that 25 patients had a squamous cell carcinoma (35.2%) and 41 adenocarcinoma (57.7%). The mean number of dissected lymph nodes was 33 (range, 10-60). All patients received postoperative radiotherapy with a mean dose of 50 Gy for R0 resection (range, 50-56 Gy) and a mean dose of 60 Gy for R1 resection (range, 40-60 Gy). The median interval between surgery and postoperative radiotherapy was 1 month (range, 1-3 months) for the group of patients receiving preoperative chemotherapy and 4 months (range 2-12 months) for the group of patients receiving postoperative chemotherapy. Patients´ characteristics are presented in Table 1.

**Table 1:** **Patients characteristics**

Clinical characteristics	Number (n)	%
Total number	71	100
Age (years)		
Median	59	
Range	29 - 75	
Gender		
Male	48	68
Female	23	32
Disease stage		
IIIA	66	93
IIIB	5	7
Tumor classification		
T1	7	10
T2	43	60
T3	16	23
T4	5	7
Lymph node status		
N2	71	100
Tumor location		
Right upper lobe	28	39
Right middle lobe/central	5	7
Right lower lobe	9	13
Left upper lobe	16	23
Left lower lobe	13	18
Tumor histology		
Squamous cell	25	35
Adenocarcinoma	41	58
Large cell	3	4
Other	2	3
Surgery type		
Lobectomy	44	62
Bi-lobectomy	7	10
Pneumonectomy	20	28
Resection status		
R0	53	75
R1	18	25
Resected lymph nodes		
Median	33	
Range	10 – 60	
Chemotherapy		
Induction	23	32
Adjuvant	48	68
RT dose (Gy)		
Median	50	
Range	40 - 60	

### Survival results

**Figure 1:**
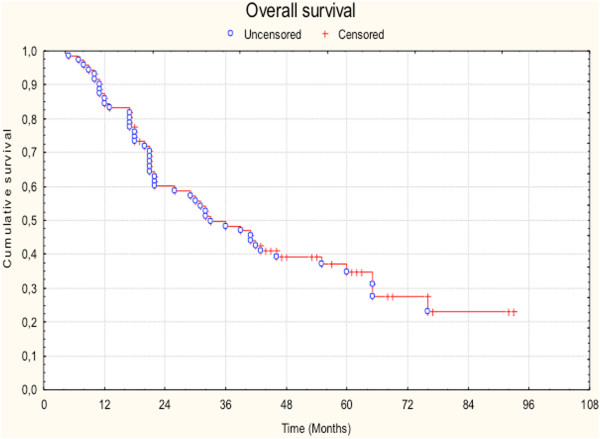
**Overall survival.**

**Figure 2:**
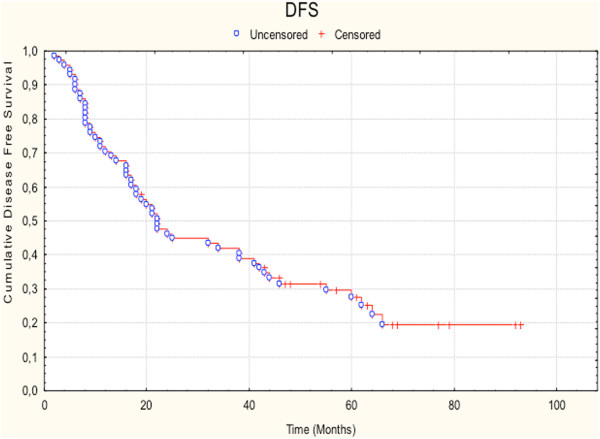
**Disease free survival.**

Overall analysis revealed a median survival time of 32 months. 1-, 3- and 5-year survival rates were 84.5%, 49.6% and 35.5% respectively. Disease-free survival rates at 1, 3 and 5 years were 70.4%, 41.8% and 27.4%. The Kaplan-Meier estimates for OS and DFS are presented in Figure 1 and Figure 2 respectively.

Investigation of differences in OS and DFS between various groups was performed using the log-rank test and the Fishers Exact test. Univariate analysis is presented in Table 2. For patients with R0 and R1 resection, the median survival time was 33 and 31 months respectively. The 1-, 3- and 5-year OS rates were 82.6%, 49.4% and 37.6% for the R0 group and 80%, 51.4% and 43.5% for the R1 group (p = 0.54). The respective values for 1-, 3- and 5-year DFS were 67.4%, 37.0% and 32.3% for R0 and 73.3%, 51.6% and 22.1% for R1 resection. A local relapse was noticed in 5 patients from the R0 group (9.4%) and 4 patients from the R1 group (22.2%).

**Table 2:** **Treatment outcome after trimodal therapy**

Univariate analysis	MST (months)	5-year survival (%)	P
Resection status			
R0	33	37.6	0.54
R1	31	43.5	
Histology			
Squamous cell	43	42.5	0.20
Adenocarcinoma	21	36.0	
Chemotherapy			
Induction	36	38.7	0.87
Adjuvant	32	38.7	
Type of surgery			
Lobectomy	43	51.9	0.054
Bi-lobe/Pneumonectomy	22	19.5	

Univariate analysis.

Survival outcome was separately investigated for patients with squamous cell carcinoma (SCC) and adenocarcinoma. Median survival was 43 months for SCC but only 21 months for adenocarcinoma. 1-, 3- and 5-years OS and DFS for SCC were 85%, 60% and 42.5%, and 80.0%, 55.0% and 34.2% respectively. For adenocarcinoma OS and DFS were 77.1%, 42.0%, 36.0% and 60.0%, 28.6% and 28.6% respectively (p = 0.20 for OS and p = 0.21 for DFS).

In regard to chemotherapy, analysis focused on the time of chemotherapy in respect to surgery (induction vs. postoperative chemotherapy). The group of patients that received induction chemotherapy had a median survival of 36 months. 1-, 3- and 5-year OS was 81.8%, 53.3% and 38.7%, whereas the respective DFS values were 68.2%, 45.5% and 31.8%. Patients who received postoperative chemotherapy showed similar results (82.0%, 48.1% and 38.7% OS rates and 69.2%, 37.8% and 28.7% DFS rates at 1, 3 and 5 years respectively). The median survival was 32 months for adjuvant chemotherapy. No significant difference was noticed (p = 0.87 for OS and p = 0.79 for PFS).

Patients who received cisplatin had a median survival of 41 months. In comparison median survival was 22 months for patients who did not receive cisplatin. 1-, 3- and 5-year OS was 83.8%, 54.1% and 40.2% for cisplatin-containing treatment and 79.2%, 43.5% and 36.8% for non-cisplatin chemotherapy.

To evaluate the role of surgical treatment for therapy outcome, overall survival was analyzed for the group of patients who received lobectomy and the group of patients who received bilobectomy or pneumonectomy. Median survival was 43 months for the lobectomy group and 22 months for the bilobectomy/pneumonectomy group. 1-, 3- and 5-years overall survival was 86.0%, 59.5% and 51.9% for the lobectomy group and 78.6%, 35.7% and 19.5% for the bilobectomy/pneumonectomy group respectively. Log rank analysis revealed that this difference just failed statistical significance (p = 0.054).

The multivariate analysis using a Cox regression model did not reveal any statistically significant independent prognostic factors for overall survival (Table 3), but indicated a trend for pT stage (HR = 1.71, 95% CI: 0.92-3.18, p = 0.088) and the type of surgery (bilobectomy/pneumonectomy versus lobectomy, HR = 2.01, 95% CI: 0.92-4.39, p = 0.078).

**Table 3:** **Multivariate analysis**

Multivariate	HR	SE	z	P > |z|	[95% conf. interval]	
Age	.99497	.01878	-0.27	0.790	.9588233	1.032488
pT	1.7132	.54108	1.70	0.088	.9225897	3.181654
Non-adenocarcinoma	.61179	.26439	-1.14	0.256	.2622703	1.427124
Bi-lob/Pneumonectomy	2.0140	.80082	1.76	0.078	.9238394	4.390594
Induction-chemotherapy	1.5673	.64774	1.09	0.277	.6972494	3.523288
Non-cisplatin	1.3349	.55323	0.70	0.486	.5925367	3.007608

### Distant metastasis

Among 71 patients initially diagnosed with stage III NSCLC, 31 patients (44%) developed distant metastases, 8 patients (11.2%) pulmonary metastases, whereas 7 patients (9.8%) developed bone metastases. The majority of the cases with distant failure (16 patients, 22.5%) were diagnosed with cerebral metastases. The median distant metastasis free survival was 13 months (range, 2-33 months). Among all patients with distant metastases 22 patients (71%) were diagnosed with adenocarcinoma, whereas only 9 patients (29%) had a non-adenocarcinoma disease (p = 0.039). The respective values for cerebral metastases were 81% (13 cases) for adenocarcinoma and 19% (3 cases) for non-adenocarcinoma histology (p = 0.028).

### Radiation toxicity

**Figure 3:**
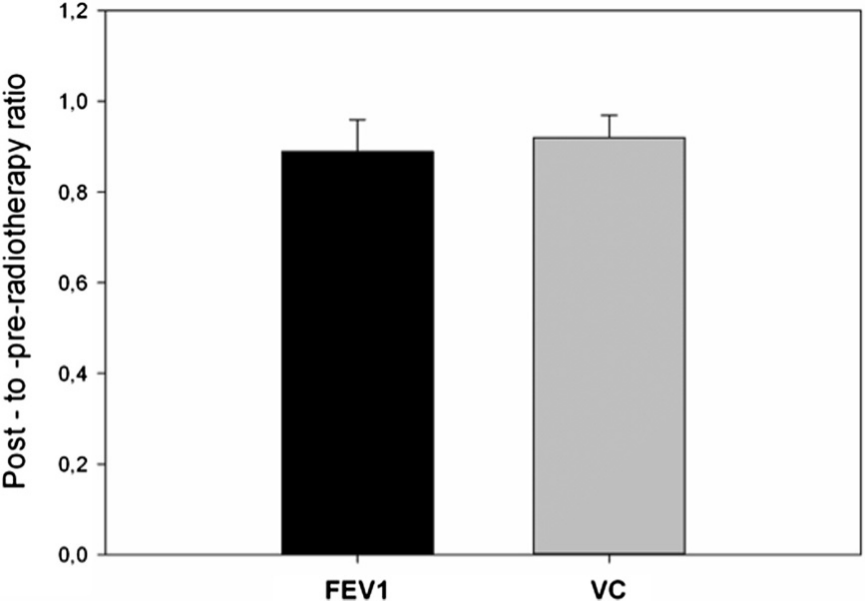
**Post- to pre-radiotherapy ratio FEV1 and VC. Mean values and standard deviation.**

Toxicity of postoperative radiotherapy within the trimodal treatment of patients with stage III non-small-cell lung cancer was investigated. Among 71 patients that were included in the analysis 6 (8.4%) developed a clinically relevant ≥ grade 2 pneumonitis. All cases developed within 120 days post radiation treatment. 4 patients (5.6%) developed a grade 3 esophagitis, whereas in 1 case (1.4%) an esophago- tracheal fistula was diagnosed. To evaluate the functional effects of mediastinal radiotherapy, pulmonary function post-surgery but pre-radiotherapy was investigated and compared to the pulmonary function 1-3 years post-radiotherapy. The post-to-pre radiotherapy ratio of FEV_1_ was 89%, whereas the same value for VC was calculated to be about 92% (Figure 3). Evaluation of the forced expiratory volume in 1 s per unit of vital capacity (FEV1/VC) revealed a median decrease post radiotherapy of 2.1%.

## Discussion

The optimal treatment for stage III non-small cell lung cancer is subject of intensive clinical research, mainly due to the fact that this stage includes patients with heterogeneous disease characteristics. Aim of the present study is to evaluate the impact of trimodal treatment in patients with stage III-N2 NSCLC. We performed a retrospective analysis of 71 cases that received surgical resection including mediastinal lympadenectomy, induction or adjuvant chemotherapy and postoperative radiation treatment. The 5-year overall and progression free survival rates were 35.5% and 27.4%. Locoregional failure after radiotherapy was 12.6%. Statistical comparisons between various groups revealed a trend for improved outcome for patients with non-adenocarcinoma histology, patients that received cisplatin-based chemotherapy and patients who underwent simple lobectomy. Toxicity analysis demonstrated > grade 2 pneumonitis and oesophagitis rates of 8.4% and 5.6% respectively and revealed that postoperative radiotherapy caused a 10% decrease of FEV1 and VC. Our study furthermore showed that 44% of the patients developed distant metastases with the majority diagnosed with cerebral metastatic disease.

Analyses of the outcome of multimodal combinatorial treatments for stage III NSCLC are of high clinical significance and relevance. Whereas the role of chemotherapy in the treatment of stage III NSCLC has been extensively investigated, the impact of postoperative radiation therapy within multimodal approaches has not been completely cleared. Based on the fact that resected stage III-N2 patients have locoregional relapse rates varying between 18% with and 29% without chemotherapy [11] the role of postoperative radiotherapy seems to be crucial. Although data clearly indicate a significant reduction in local recurrence after postoperative radiotherapy for N2 NSCLC, the survival effects remain controversial [15–17].

Our study revealed a median OS of 32 months and a 5-year OS rate of 35.5% for the trimodal treatment, which seems to be higher compared to the survival rates of the SEER analysis (27% 5-year OS) [15]. However, the SEER database evaluation did not include data on the use of chemotherapy, which impedes a comparison to our data. On the contrary in comparison to the results of the ANITA trial our results seem on a first view to be inferior. In particular, within the ANITA trial patients with N2 disease who received a postoperative radiation therapy had a median survival of 47.4 months and a 5-year survival of 47.4% in the chemotherapy group [9]. The discrepancy between the ANITA trial and our study is minimized when the chemotherapy scheme is included in the analysis. Considering that patients in ANITA received cisplatin-based chemotherapy, analysis of our data showed that the cisplatin-based chemotherapy group had improved survival (median 41 months, 5-year OS rate 40.2%), which was comparable to the outcome of the ANITA trial.

Most reports on trimodality strategies for stage III-N2 NSCLC involve trials of preoperative radiochemotherapy. Prominent examples of phase III randomized controlled trials are the INT 0139/RTOG 9309 [4] and the German Cancer Cooperative Group study [10]. Within the INT 0139 study 216 patients with N2-lymph node status received induction chemotherapy plus radiotherapy to a dose of 45 Gy followed by surgery. Median survival and 5-year survival were 23.6 months and 27% respectively. Our retrospective data indicate improved survival, which might be attributed to the fact that in the INT0139 study patients received preoperative radiation treatment. A negative impact of preoperative radiation therapy has been revealed in the GLCCG trial. Thomas et al. showed that treatment related mortality was increased when radiotherapy was applied prior to surgery, an effect that was stronger for the group of patients who received pneumonectomy [10]. Lower 5-year survival rates were reported in more recent trials of a trimodality regimen with neoadjuvant radiochemotherapy (21.7%), however the majority of the patients in the study by Friedel et al. had stage IIIB disease [18]. A multicentric phase II trial by Stupp et al. revealed a median overall survival of 29 months and 5-year survival rate of 40% for patients with stage IIIB NSCLC who received neoadjuvant chemotherapy and radiotherapy followed by surgery [19]. In addition, recently the phase II trial CISTAXOL reported 10-year long-term survival of 26% [20]. These data indicate that despite the fact that definitive radiochemotherapy is preferred for stage IIIB disease [21], trimodal treatment including surgery should be considered for cases that are technically resectable.

In regard to the role of postoperative radiotherapy on locoregional control our data revealed a local relapse rate of 12.6% for the entire patient cohort and 9.4% for patients after R0 resection. These rates are higher compared to the results of a retrospective analysis of the ANITA trial, which revealed locoregional relapse in 6.3% of the patients with pN2 who were randomized to chemotherapy and received postoperative radiotherapy. However the local relapse rates for the entire population in the ANITA trial were 12% in the chemotherapy group and 18% in the observation group [9]. Considering that the ANITA trial included also patients with lower disease stages (IB-IIIA) and that about 22% of the patients in the chemotherapy arm received additional postoperative radiotherapy higher local relapse rates are expected for stage IIIA patients without radiation treatment. This is also supported by data from studies in which patients received only chemotherapy and surgery. In particular chemotherapy followed only by surgery revealed local failure rates of about 23.6-29% [22, 23]. Despite limitations, a comparison of our data to these results indicates a superior local control for the trimodal treatment.

No survival difference between induction and postoperative chemotherapy was shown in our analysis. This result seems to be in concert with meta-analyses indicating an absolute 5-year survival benefit of about 5% for neoadjuvant chemotherapy [24, 25]. Despite limitations in comparing the data of meta-analyses on pre- and postoperative chemotherapy, the benefit of preoperative chemotherapy seems to be similar to the benefit of adjuvant approaches, allowing the suggestion that the relative effects of neoadjuvant and adjuvant systemic therapy are comparable. However, data indicate a higher compliance for preoperative chemotherapy (about 90%), compared to postoperative treatment (about 60%) [25, 26]. Although safe conclusions are limited by the fact that such data are provided from studies with more early disease stages [26], still, they reveal that determination of the optimal therapy sequence is of high importance and needs to be further evaluated in prospective, randomized, clinical trials applying multimodal treatment. In addition to the therapy sequence, we further investigated whether cisplatin-based chemotherapy was associated with improved therapy outcome. Indeed, median survival was 41 months for cisplatin-based and 22 months for non-cisplatin-based chemotherapy, whereas 5-year survival rates were 40.2% and 36.8% respectively. This trend for improved outcome seems to be in concert with previous meta-analyses indicating a survival advantage for cisplatin-based adjuvant treatment [27]. More recent analyses of clinical trials in patients with advanced NSCLC show higher response rates for cisplatin in combination with third generation drugs, but not a benefit in overall survival [28]. Therefore, the trend for improved survival for cisplatin-based regimens must be interpreted with great caution, mainly because patients that receive cisplatin-based chemotherapy are characterized by better performance status and less comorbidities, which facilitates selection bias of the results.

Interestingly, patients with adenocarcinoma showed in our study a trend to poorer outcome, compared to patients with SCC. Although adenocarcinoma is known to be a potentially poor prognostic factor in patients with resected NSCLC, the addition of chemotherapy revealed in previous studies an increased benefit for this histological subtype, allowing the hypothesis that this parameter may be a predictive factor for enhanced multimodal treatment benefit [29]. A possible explanation for the survival difference in our analysis might be the significant difference in distant failure rates between the group of patients with adenocarcinoma and non-adenocarcinoma histology. Especially in regard to cerebral metastases, previous studies have confirmed the correlation between brain progression and histological features [30, 31], raising again the question of a possible benefit through prophylactic cranial irradiation (PCI). A recent study reassessing the potential of PCI in the current era of multimodal NSCLC treatment, demonstrated that PCI decreases the rate of brain metastases but does not seem to improve overall survival [32]. Although PCI is not recommended as standard therapy in NSCLC on the basis of this data, still further studies focusing on high risk patients, including adenocarcinoma need to be performed. In this direction a new multidisciplinary classification of adenocarcinomas, based on pathologic, molecular and radiologic features is expected to facilitate the improvement of patient stratification [33].

Both univariate and multivariate analysis indicated that the type of surgery might have an influence on treatment outcome. In particular, bi-lobectomy/pneumonectomy was associated with a strong trend to a decreased survival compared to lobectomy. This result is in concert with further studies showing that lobectomy was marginally associated with a higher overall survival rate compared to pneumonectomy [34] and can be logically explained by the fact that patients who are stratified to bilobectomy or pneumonectomy have usually tumors with disadvantageous characteristics, such as larger size and/or infiltration of central structures. Similar to previous analyses the type of surgery was not found in our analysis to be of prognostic significance. Considering however the fact that the p value was very close to the significant level (p < 0.05), this might be attributed to the relative low number of patients included in our study. Therefore, further investigation is necessary in order to generate safe conclusions. However, when lobectomy/bi-lobectomy was compared to pneumonectomy there was no significant difference (p = 0.3), a result which is in concert with further recent studies, indicating that pneumonectomy can be done safe and may not be a risk factor for survival in trimodal therapy of stage III NSCLC [35].

A major drawback in the use of multimodal therapeutic approaches for NSCLC is the treatment-related toxicity. In this respect post-operative radiotherapy is mostly associated with pneumonitis. A study focusing on radiation-induced lung injury for postoperative radiotherapy and the impact of pre-radiotherapy surgery on it revealed a symptomatic pneumonitis rate of about 19%, which was similar in surgical and non-surgical groups [36]. Further analyses revealed that pneumonitis occurs in 1-28% of lung cancer patients treated with postoperative radiotherapy [37], with the irradiated volume of healthy lung parenchyma known to be the most important risk factor. However the results between the various trials cannot be directly compared due to a large heterogeneity in the patient collectives. In our analysis clinically relevant > grade 2 pneumonitis was noticed in 8.4% of all cases. This might be associated with the fact that only low volumes of lung parenchyma were irradiated since target definition included in most cases only the mediastinal lymphatics. However, a recent phase II trial on neoadjuvant chemoradiation for stage III NSCLC revealed comparable results (8.5% grade 2 pneumonitis) [38]. A further issue associated with radiation toxicity in NSCLC refers to alterations in pulmonary function, which is mostly observed as a decrease in diffusion capacity and FEV1. Whereas older studies have shown an FEV1 decrease of about 10-20% [39], our analysis demonstrated a decrease of about 11% for FEV1 after adjuvant radiotherapy within multimodal approaches. Furthermore, a decrease in the ratio FEV1/VC of about 2.1% was calculated in our study. In comparison, a recent analysis of 250 patients who had received ≥60 Gy radiotherapy for primary NSCLC showed a decrease in the median FEV1/VC level after radiotherapy of 3.7% 9-12 months post treatment [40]. It should be mentioned, however, that the lung function might be influenced by the time interval between surgery and radiotherapy, considering that thoracic surgery can result in both a permanent decrease of lung function due to resection of pulmonary parenchyma, and a temporary reduction due to reversible tissue changes on the remaining parenchyma. Since there are different medians in the time between surgery and postoperative radiotherapy for the neoadjuvant and the adjuvant setting, we performed the analyses for the 2 settings separately. For patients receiving adjuvant chemotherapy (median time between surgery and RT 4 months) the post-to-pre radiotherapy ratio of FEV_1_ was 90%, whereas the same value for VC was 92%. For the group of patients receiving neoadjuvant chemotherapy (median time between surgery and RT 1 month) the respective values were 87% and 91%. Despite the differences in the time between surgery and radiotherapy between the 2 groups the differences in%FEV1 and VC were not statistically significant in our study. Still, the intervall between resection and radiotherapy is very important and should be critically considered in further analyses, focussing on the pulmonary function after multimodal therapeutic approaches.

## Conclusions

In conclusion, the present descriptive data indicate that trimodal combinatorial therapy represents an effective and safe treatment approach for patients with resectable stage III-N2 non-small-cell lung cancer. Addition of postoperative radiotherapy to the established combination of surgery and chemotherapy facilitates the improvement of locoregional control. Although our results are mostly in concert with previous trials and further related studies, critical limitations need to be considered. Beside the retrospective character of our work, which might facilitate selection bias, the included number of patients might not allow safe statistical conclusions, emphasizing the necessity for cautious interpretations. Therefore, further analyses of larger patient collectives as well as prospective clinical trials are necessary in order to clearly define the impact of postoperative radiotherapy within multimodal therapeutic strategies for stage III disease, identify patients with improved benefit/risk ratios and optimize treatment of non-small-cell lung cancer.
